# Evolution and Comparative Genomics of F33:A−:B− Plasmids Carrying *bla*_CTX-M-55_ or *bla*_CTX-M-65_ in *Escherichia coli* and *Klebsiella pneumoniae* Isolated from Animals, Food Products, and Humans in China

**DOI:** 10.1128/mSphere.00137-18

**Published:** 2018-07-18

**Authors:** Jing Wang, Zhen-Ling Zeng, Xin-Yi Huang, Zhen-Bao Ma, Ze-Wen Guo, Lu-Chao Lv, Ying-Bi Xia, Li Zeng, Qian-Hua Song, Jian-Hua Liu

**Affiliations:** a College of Veterinary Medicine, Key Laboratory of Zoonosis of Ministry of Agricultural and Rural Affairs, South China Agricultural University, Guangzhou, China; Escola Paulista de Medicina/Universidade Federal de São Paulo

**Keywords:** IncFII, antimicrobial resistance, expanded-spectrum β-lactamases, IncFII

## Abstract

To understand the underlying evolution process of F33:A−:B− plasmids among *Enterobacteriaceae* isolates of various origins in China, the complete sequences of 17 *bla*_CTX-M_-harboring F33:A−:B− plasmids obtained from Escherichia coli and Klebsiella pneumoniae isolates from different sources (animals, animal-derived food, and human clinics) in China were determined. F33:A−:B− plasmids shared similar plasmid backbones comprising replication, leading, and conjugative transfer regions and differed by the numbers of repeats in *yddA* and *traD* and by the presence of group II intron, except that pHNAH9 lacked a large segment of the leading and transfer regions. The variable regions of F33:A−B− plasmids were distinct and were inserted downstream of the addiction system *pemI*/*pemK*, identified as the integration hot spot among F33:A−B− plasmids. The variable region contained resistance genes and mobile elements or contained segments from other types of plasmids, such as IncI1, IncN1, and IncX1. Three plasmids encoding CTX-M-65 were very similar to our previously described pHN7A8 plasmid. Four CTX-M-55-producing plasmids contained multidrug resistance regions related to that of F2:A−B− plasmid pHK23a from Hong Kong. Five plasmids with IncN and/or IncX replication regions and IncI1-backbone fragments had variable regions related to those of pE80 and p42-2. The remaining five plasmids with IncN replicons and an IncI1 segment also possessed closely related variable regions. The diversity in variable regions was presumably associated with rearrangements, insertions, and/or deletions mediated by mobile elements, such as IS*26* and IS*1294*.

**IMPORTANCE** Worldwide spread of antibiotic resistance genes among *Enterobacteriaceae* isolates is of great concern. F33:A−:B− plasmids are important vectors of resistance genes, such as *bla*_CTX-M-55/-65_, *bla*_NDM-1_, *fosA3*, and *rmtB*, among E. coli isolates from various sources in China. We determined and compared the complete sequences of 17 F33:A−:B− plasmids from various sources. These plasmids appear to have evolved from the same ancestor by mobile element-mediated rearrangement, acquisition, and/or loss of resistance modules and similar IncN1, IncI1, and/or IncX1 plasmid backbone segments. Our findings highlight the evolutionary potential of F33:A−:B− plasmids as efficient vectors to capture and diffuse clinically relevant resistance genes.

## Abstract (precis)

Worldwide spread of antibiotic resistance genes among *Enterobacteriaceae* isolates is of great concern. F33:A−:B− plasmids are important vectors of resistance genes, such as *bla*_CTX-M-55/-65_, *bla*_NDM-1_, *fosA3*, and *rmtB*, among E. coli isolates from various sources in China. We determined and compared the complete sequences of 17 F33:A−:B− plasmids from various sources. These plasmids appear to have evolved from the same ancestor by mobile element-mediated rearrangement, acquisition, and/or loss of resistance modules and similar IncN1, IncI1, and/or IncX1 plasmid backbone segments. Our findings highlight the evolutionary potential of F33:A−:B− plasmids as efficient vectors to capture and diffuse clinically relevant resistance genes.

## INTRODUCTION

Plasmids are crucial vehicles for worldwide spread of antibiotic resistance genes in Gram-negative bacteria. Several plasmid families, such as IncF, IncI1, IncI2, IncX, IncA/C, and IncHI2, play an important role in the global dissemination of expanded-spectrum β-lactamase genes, AmpC β-lactamase genes, carbapenemase genes, plasmid-mediated quinolone resistance genes, and colistin resistance gene *mcr-1* in *Enterobacteriaceae* isolates (1–3).

F33:A−:B− plasmids are some of the most predominant replicon sequence types (RSTs) among IncF multiresistance plasmids from Escherichia coli isolates of animal origin in China (4). Our previous studies confirmed that F33:A−:B− plasmids were major vehicles for *fosA3*-*bla*_CTX-M-65_-*rmtB* transmission among food animals and pets in Guangdong Province, China (5, 6); similar F33:A−:B− plasmids associated with *rmtB*-*bla*_CTX-M-65_ were disseminated in pets in southern China and in a pig farm and its environment in eastern China (7, 8). In addition, F33:A−:B− or IncN-F33:A−:B− plasmids were responsible for the dissemination of *fosA3* and *bla*_CTX-M-55/-65_ genes in E. coli from chickens in China and pigs, chickens, and dairy cows in northeast China (9–11). Furthermore, *oqxAB* colocated with *bla*_CTX-M-55_ on F33:A−:B− and IncN-F33:A−:B− plasmids was identified in food-producing animals, chicken meat, and humans in China (12–15). Recently, F33:A−:B− plasmids were also described as carriers of *bla*_NDM-1_ from porcine E. coli isolates in China (16). Interestingly, two plasmids, p397Kp and p477Kp, which are highly similar to our previously sequenced E. coli F33:A−:B− pHN7A8 plasmid (*bla*_CTX-M-65_, *fosA3*, and *rmtB*) collected from a dog (17), were identified in clinical Klebsiella pneumoniae isolates from the Bolivian Chaco region (18). Taken together, previous studies suggest that F33:A−:B− plasmids involved in the spread of *bla*_CTX-M_, *bla*_NDM_, *fosA3*, *rmtB*, and *oqxAB* have been efficiently and widely disseminated in E. coli strains of various origins, particularly animals, in China (see Table S1 in the supplemental material).

10.1128/mSphere.00137-18.2 TABLE S1  Characteristics of *Enterobacteriaceae* carrying F33:A−:B− plasmids from animals, food, human clinics, and the environment. Download TABLE S1, PDF file, 0.4 MB. Copyright © 2018 Wang et al. 2018 Wang et al. This content is distributed under the terms of the Creative Commons Attribution 4.0 International license.

Here, we aimed to determine and compare the complete sequences of 17 F33:A−:B− plasmids harboring *bla*_CTX-M_ obtained from E. coli and K. pneumoniae isolates from different sources (animals, animal-derived food products, and human clinics), providing new insights into the evolution of F33:A−:B− plasmids among *Enterobacteriaceae* isolates of various origins in China.

## RESULTS AND DISCUSSION

### Strains and F33:A−:B− resistance plasmids.

Complete nucleotide sequences were determined for 17 F33:A−:B− transmissible plasmids obtained from 15 E. coli isolates from food-producing animals, food, and patients and 2 K. pneumoniae isolates from pork (Table 1). The plasmids ranged in size between 55,683 and 145,804 bp and contained 2 to 11 resistance genes (Table 1 and 2). Eleven E. coli isolates carrying F33:A−:B− plasmids subjected to multilocus sequence typing (MLST), either previously (9, 14) or in this study, were identified as 11 different sequence types (STs), and two K. pneumoniae isolates belonged to ST35. They were distinct from previously described isolates carrying F33:A−:B− resistance plasmids (see Table S1 in the supplemental material), further indicating the important role of F33:A−:B− plasmids in the horizontal transfer of resistance genes between bacteria and in the adaptation of these plasmids to different hosts with genomic differences (Table 1).

**TABLE 1:** General features of F33:A−:B− plasmids analyzed in this study and of related reference plasmids for comparative analysis^a^

Strain	Species	MLST^b^	Plasmid	Size (bp)	GenBank accession no.	Location	Yr of isolation	Isolate origin	Reference or source
HN7A8*	E. coli	ND	pHN7A8	76,878	JN232517	Guangdong Province, China	2008	Dog	17
FKP460#	E. coli	354	**pHNFP460-1**	99,868	KJ020575	Guangdong Province, China	2010	Pig	29
04NHB3	E. coli	ND	**pHN04NHB3**	104,623	MG197488	Guangdong Province, China	2009	Duck	29
MC02#	E. coli	2732	**pHNMC02**	70,619	MG197489	Guangdong Province, China	2009	Chicken	29
FKD271#	E. coli	ND	**pHNFKD271**	104,703	MG197490	Guangdong Province, China	2010	Duck	29
FKU92#	E. coli	ND	**pHNFKU92**	109,185	MG197491	Guangdong Province, China	2013	Duck	This study
GDK4P177#	E. coli	ND	**pHNGD4P177**	70,643	MG197492	Guangdong Province, China	2014	Pig	This study
AHC9	E. coli	48	**pHNAH9**	55,683	MG197493	Anhui Province, China	2011	Chicken	9
AHC17	E. coli	4483	**pHNAH17**	96,376	MG197494	Anhui Province, China	2011	Chicken	9
AHC24	E. coli	155	**pHNAH24**	145,804	MG197495	Anhui Province, China	2011	Chicken	9
AHC33	E. coli	101	**pHNAH33**	74,962	MG197496	Anhui Province, China	2011	Chicken	9
HNC02	E. coli	4464	**pHNHNC02**	76,869	MG197497	Henan Province, China	2009	Chicken	9
HZMCC14*	E. coli	1290	**pHNMCC14**	142,896	MG197498	Guangdong Province, China	2011	Chicken meat	This study
HZMPC32*	E. coli	New	**pHNMPC32**	74,768	MG197499	Guangdong Province, China	2011	Pork	This study
HZMPC51-2*	K. pneumoniae	35	**pHNMPC51**	69,654	MG197500	Guangdong Province, China	2011	Pork	This study
HZMPC43-3*	K. pneumoniae	35	**pHNMPC43**	69,666	MG197501	Guangdong Province, China	2011	Pork	This study
ZYTF32*	E. coli	58	**pHNZY32**	145,804	MG197502	Guangdong Province, China	2013	Patient	14
ZYTM118*	E. coli	New	**pHNZY118**	145,804	MG197503	Guangdong Province, China	2013	Patient	14
397Kp	K. pneumoniae	726	p397Kp	76,863	LN897474	Bolivia	2013	Patient	18
477Kp	K. pneumoniae	726	p477Kp	74,768	LN897475	Bolivia	2013	Patient	18
HNEC55	E. coli	1721	pHNEC55	81,498	KT879914	Henan Province, China	2014 or 2015	Pig	16
CH292B	E. coli	10	pECB11	92,545	KY865321	Jilin Province, China	2015 or 2016	Chicken	10
42–2*	E. coli	NA	p42-2	106,886	KT990220	Guangdong Province, China	2010	Duck	15
E80△	E. coli	NA	pE80	138,718	KU321583	Guangdong Province, China	2013	Chicken meat	13
SLK172	E. coli	189	pSLK172-2	120,528	CP017633	Beijing, China	2015	Patient	35

a Plasmids with names in bold typeface were sequenced in this study. Abbreviations: NA, not available; ND, not determined. Strains isolated from Guangzhou, Foshan, or Shenzhen are indicated by an asterisk, pound sign, or triangle.

b The HZMPC32 isolate was identified as a new MLST with alleles *adk457*, *fumC65*, *gyrB5*, *icd16*, *mdh11*, *purA8*, and *recA6*. The ZYTM118 isolate was previously identified as a new MLST with alleles *adk64*, *fumC23*, *gyrB358*, *icd91*, *mdh307*, *purA7*, and *recA2* (13).

### The backbone sequences of F33:A−:B− plasmids are highly conserved.

**FIG 1:**
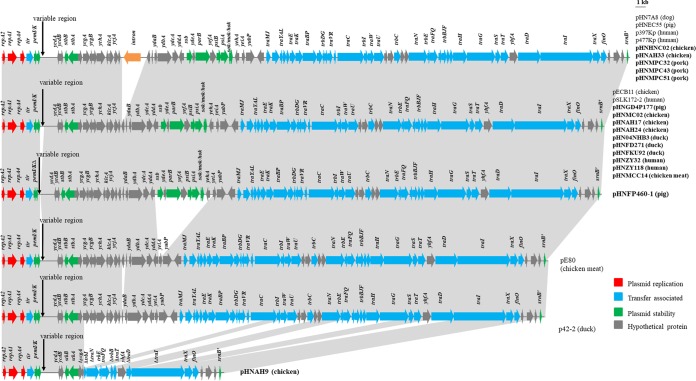
Linear comparisons of F33:A−:B− plasmid backbones. Regions of over 99% homology are shaded in gray. Labeled vertical arrows indicate the insertion points of variable regions that were removed. Plasmids with names in bold typeface were sequenced in this study. The sequences used to draw these diagrams are from the GenBank accession numbers listed in Table 1.

**FIG 2:**
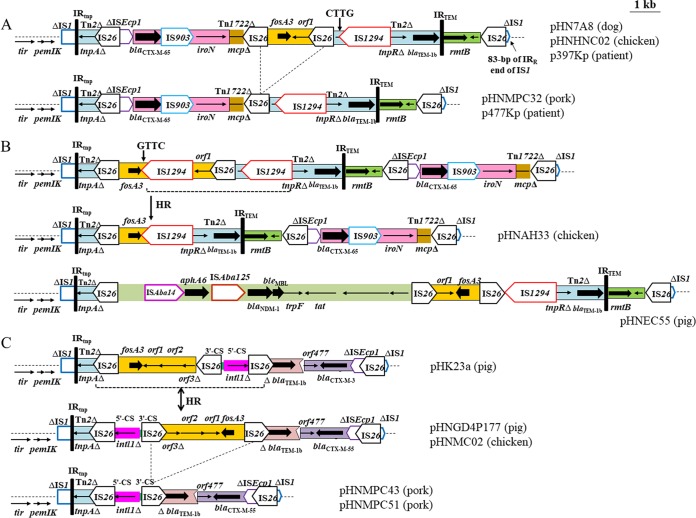
Comparison of related MRRs in (A) pHNHNC02, pHNMPC32, pHN7A8, p477Kp, and p397Kp; (B) pHNAH33 and pHNEC55; and (C) pHNGD4P177, pHNMC02, pHNMPC43, pHNMPC51, and pHK23a. The extents and directions of antibiotic resistance genes (thick arrows) and other genes are indicated. Insertion sequences (ISs) are shown as boxes labeled with the IS name. Tall bars represent the 38-bp TIR of transposons. The backbone is indicated by dotted lines. Arrows labeled with "HR" and dotted lines indicate where homologous recombination could explain differences between structures. Dotted diagonal lines indicate possible deletion and insertion events. Target sites of IS*1294* are labeled with black arrows. Diagrams were drawn from sequences from the GenBank accession numbers listed in Table 1 plus sequences from plasmid pHK23a and GenBank accession number JQ432559.

**FIG 3:**
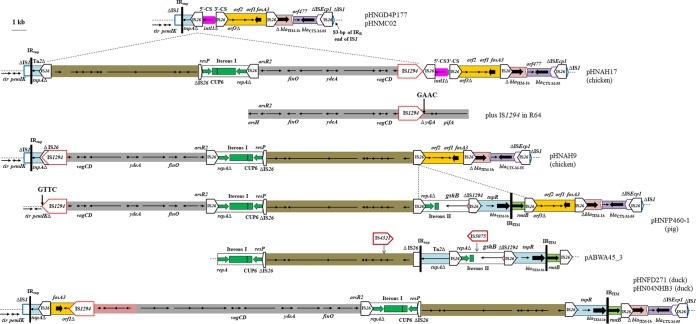
Comparison of pHNGD4P177/pHNMC02 MRR with variable regions in pHNAH9, pHNAH17, pHNFP460-1, pHNFD271, and pHN04NHB3 and relationship between them and IncI1 R64 and IncN plasmid pABWA45_3. The extents and directions of antibiotic resistance in antibiotic resistance genes (thick arrows) and in other genes are indicated. ISs are shown as boxes labeled with the IS name. Labeled vertical arrows with IS boxes indicate the insertion sites of IS elements. Tall bars represent the 38-bp TIR of transposons. The backbone is indicated by dotted lines. Dotted diagonal lines indicate possible deletion and insertion events. Target sites of IS*1294* are labeled with black arrows. Sequences referred to in this diagram are from the GenBank accession numbers listed in Table 1 plus R64, GenBank accession number AP005147, pABWA45_3, and GenBank accession number CP022157.

**FIG 4:**
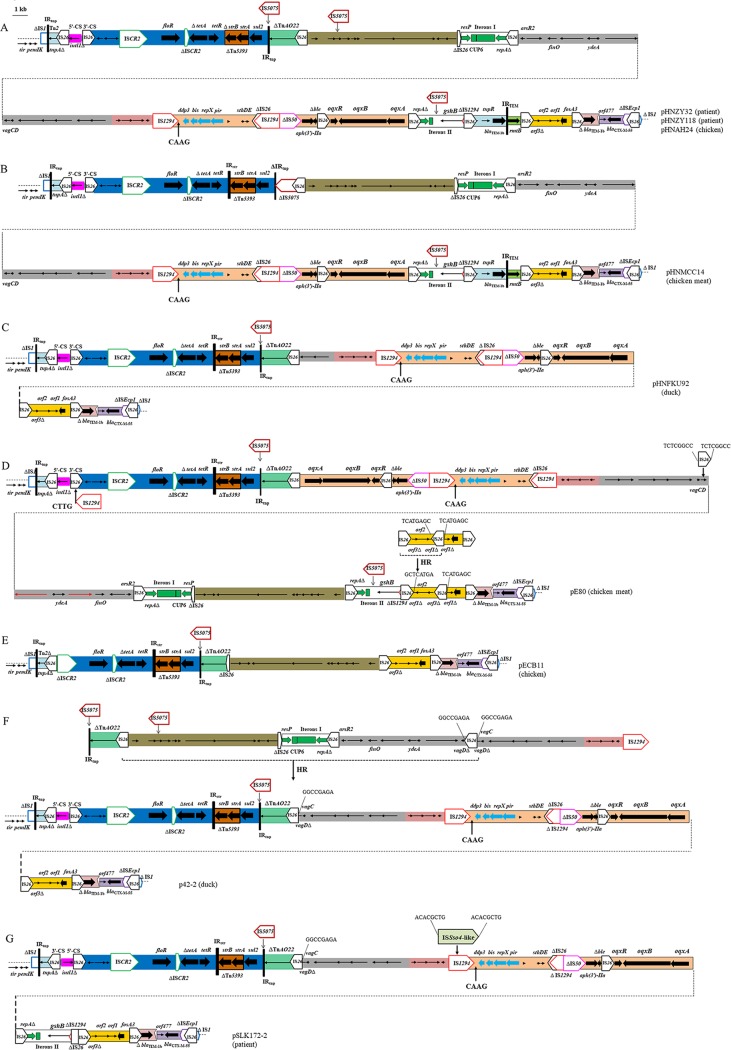
Comparison of the variable regions of (A) pHNZY32, pHNZY118, and pHNAH24; (B) pHNMCC14; (C) pHNFKU92; (D) pE80; (E) pECB11; (F) p42-2; and (G) pSLK172-2. The extents and directions of antibiotic resistance in antibiotic resistance genes (thick arrows) and in other genes are indicated. Genes in pE80 (D) could not be annotated because of nucleotide changes, and deletions are indicated by red arrows. ISs are shown as boxes labeled with the IS name. Labeled vertical arrows with IS boxes indicate the insertion sites of IS elements. Direct repeats are indicated by arrows and sequence. Tall bars represent the 38-bp TIR of transposons. The backbone is indicated by dotted lines. Arrows labeled with "HR" and dotted lines indicate where homologous recombination could explain differences between structures. Target sites of IS*1294* are labeled with black arrows. Adjacent regions are connected with dotted lines. Sequences referred to in the diagram are from the GenBank accession numbers listed in Table 1.

All plasmids possessed the same overall backbone organization as F33:A−:B− plasmid pHN7A8, corresponding to replication, leading, and transfer regions, with the exception of pHNAH9 (Fig. 1). The replication regions of all 17 plasmids (*repA2*/*copB*-*repA1-repA4*) were identical to those of many F33:A−:B− plasmids. The leading region contained genes related to plasmid maintenance and stability, such as *pemI*/*pemK*, *stbA*/*stbB*, and *sok*/*hok*/*mok*, and showed 99% identity to many F33:A−:B− plasmids, but a large segment of the leading region was absent in pHNAH9, which might have been the result of a recombination event between *ycgA* and *trbI*. The integration hot spot among F33:A−:B− plasmids was identified downstream of the addiction system *pemI*/*pemK*, in which the variable region comprising backbone segments from other plasmid types and/or resistance modules was inserted and bounded at both ends by fragments of IS*1*, with the exception that *pemK* was truncated by IS*1294* in pHNFP460-1 (Fig. 2, 3, and 4). In addition, five plasmids in this study and plasmid pHN7A8 carried a putative group II intron inserted downstream of the *ycjA* gene, as identified in pHNEC55, p397Kp, and p477Kp but not pECB11, pSLK172-2, p42-2, and pE80 (Table 2). Furthermore, different numbers of 6-bp tandem repeats (GCTACT) in *yddA* were present in F33:A−:B− plasmids (Table 2). The transfer regions of plasmids were highly similar to those of plasmid pHN7A8 and other F33:A−:B− plasmids and differed by the numbers of CAACAGCCG tandem repeats in the *traD* gene (Table 2). However, possibly due to multiple recombination events, pHNAH9 lacked large parts of the *tra*-*trb* region, which might account for its conjugation failure (9).

**TABLE 2:** Characteristics of F33:A−:B− plasmids analyzed in this study

Plasmid	Resistance genes	Replicon type	No. of 6-bp repeats in *yddA*	No. of 9-bp repeats in *traD*	No. of 37-bp repeats in iteron I (IncN)	Group II intron	Addiction systems
pHN7A8	*bla*_CTX-M-65_, *bla*_TEM-1b_, *fosA3*, *rmtB*	F33:A−:B−	8	9		+	*pemKI*, *hok*-*sok*, *srnBC*
pHNFP460-1	*bla*_CTX-M-55_, *bla*_TEM-1b_, *fosA3*, *rmtB*	N1-F33:A−:B−	7	9	30	−	Δ*pemKI*, *hok*-*sok*, *vagCD*, *srnBC*
pHN04NHB3	*bla*_CTX-M-55_, *bla*_TEM-1b_, *fosA3*, *rmtB*	N1-F33:A−:B−	8	11	31	−	*pemKI*, *hok*-*sok*, *vagCD*, *srnBC*
pHNMC02	*bla*_CTX-M-55_, Δ*bla*_TEM-1b_, *fosA3*	F33:A−:B−	7	11		−	*pemKI*, *hok*-*sok*, *srnBC*
pHNFKD271	*bla*_CTX-M-55_, *bla*_TEM-1b_, *fosA3*, *rmtB*	N1-F33:A−:B−	8	11	31	−	*pemKI*, *hok*-*sok*, *vagCD*, *srnBC*
pHNFKU92	*bla*_CTX-M-55_, Δ*bla*_TEM-1b_, *floR*, Δ*tetA-tetR*, *strA*, *strB*, *sul2*, *aph(3′)-IIa*, *oqxAB*, *fosA3*, *rmtB*	X1-F33:A−:B−	7	11		−	*pemKI*, *hok*-*sok*, *stbDE*, *srnBC*
pHNGD4P177	*bla*_CTX-M-55_, Δ*bla*_TEM-1b_, *fosA3*	F33:A−:B−	8	13		−	*pemKI*, *hok*-*sok*, *srnBC*
pHNAH9	*bla*_CTX-M-55_, Δ*bla*_TEM-1b_, *fosA3*	N1-F33:A−:B−			31	−	*pemKI*, *vagCD*, *srnBC*
pHNAH17^a^	*bla*_CTX-M-55_, Δ*bla*_TEM-1b_, *fosA3*	N1-F33:A−:B−	7	11	31	−	*pemKI*, *hok*-*sok*, *vagCD*, *srnBC*
pHNAH24	*bla*_CTX-M-55_, *bla*_TEM-1b_, *floR*, Δ*tetA-tetR*, *strA*, Δ*strB*, *sul2*, *aph(3′)-IIa*, *oqxAB*, *fosA3*, *rmtB*	N1-X1-F33:A−:B−	7	11	31	−	*pemKI*, *hok*-*sok*, *vagCD*, *stbDE*, *srnBC*
pHNAH33	*bla*_CTX-M-65_, *bla*_TEM-1b_, *fosA3*, *rmtB*	F33:A−:B−	8	11		+	*pemKI*, *hok*-*sok*, *srnBC*
pHNHNC02^b^	*bla*_CTX-M-65_, *bla*_TEM-1b_, *fosA3*, *rmtB*	F33:A−:B−	8	8		+	*pemKI*, *hok*-*sok*, *srnBC*
pHNMCC14	*bla*_CTX-M-55_, *bla*_TEM-1b_, *floR*, Δ*tetA-tetR*, *strA*, *strB*, *sul2*, *aph(3′)-IIa*, *oqxAB*, *fosA3*, *rmtB*	N1-X1-F33:A−:B−	6	11	31	−	*pemKI*, *hok*-*sok*, *vagCD*, *stbDE*, *srnBC*
pHNMPC32	*bla*_CTX-M-65_, *bla*_TEM-1b_, *rmtB*	F33:A−:B−	7	8		+	*pemKI*, *hok*-*sok*, *srnBC*
pHNMPC51	*bla*_CTX-M-55_, Δ*bla*_TEM-1b_	F33:A−:B−	7	12		+	*pemKI*, *hok*-*sok*, *srnBC*
pHNMPC43	*bla*_CTX-M-55_, Δ*bla*_TEM-1b_	F33:A−:B−	9	12		+	*pemKI*, *hok*-*sok*, *srnBC*
pHNZY32	*bla*_CTX-M-55_, *bla*_TEM-1b_, *floR*, Δ*tetA-tetR*, *strA*, Δ*strB*, *sul2*, *aph(3′)-IIa*, *oqxAB*, *fosA3*, *rmtB*	N1-X1-F33:A−:B−	7	11	31	−	*pemKI*, *hok*-*sok*, *vagCD*, *stbDE*, *srnBC*
pHNZY118	*bla*_CTX-M-55_, *bla*_TEM-1b_, *floR*, Δ*tetA-tetR*, *strA*, Δ*strB*, *sul2*, *aph(3′)-IIa*, *oqxAB*, *fosA3*, *rmtB*	N1-X1-F33:A−:B−	7	11	31	−	*pemKI*, *hok*-*sok*, *vagCD*, *stbDE*, *srnBC*
p397Kp	*bla*_CTX-M-65_, *bla*_TEM-1b_, *fosA3*, *rmtB*	F33:A−:B−	7	8		+	*pemKI*, *hok*-*sok*, *srnBC*
p477Kp	*bla*_CTX-M-65_, *bla*_TEM-1b_, *rmtB*	F33:A−:B−	7	8		+	*pemKI*, *hok*-*sok*, *srnBC*
pHNEC55	*bla*_NDM-1_, *bla*_TEM-1b_, *ble*_MBL_, *aphA6*, *fosA3*, *rmtB*	F33:A−:B−	7	8		+	*pemKI*, *hok*-*sok*, *srnBC*
pECB11	*bla*_CTX-M-55_, Δ*bla*_TEM-1b_, *floR*, Δ*tetA-tetR*, *strA*, *strB*, *sul2*, *fosA3*	F33:A−:B−	9	11		−	*pemKI*, *hok*-*sok*, *srnBC*
p42-2	*bla*_CTX-M-55_, Δ*bla*_TEM-1b_, *floR*, Δ*tetA-tetR*, *strA*, *strB*, *sul2*, *aph(3′)-IIa*, *oqxAB*, *fosA3*	X1-F33:A−:B−	6	6		−	*pemKI*, Δ*vagCD*, *stbDE*, *srnBC*
pE80^c^	*bla*_CTX-M-55_, Δ*bla*_TEM-1b_, *floR*, Δ*tetA-tetR*, *strA*, *strB*, *sul2*, *aph(3′)-IIa*, *oqxAB*, *fosA3*, *rmtB*	N1-X1-F33:A−:B−	8	8	28	−	*pemKI*, Δ*vagCD*, *stbDE*, *srnBC*
pSLK172-2	*bla*_CTX-M-55_, Δ*bla*_TEM-1b_, *floR*, Δ*tetA-tetR*, *strA*, *strB*, *sul2*, *aph(3′)-IIa*, *oqxAB*, *fosA3*	X1-F33:A−:B−	6	4		−	*pemKI*, *hok*-*sok*, Δ*vagCD*, *stbDE*, *srnBC*

a pHNAH17 was previously detected to carry *rmtB* by PCR (9), but plasmid sequencing and further PCR in this study demonstrated that *rmtB* was not present.

b pHNHNC02 was formerly detected to harbor *vagCD* by PCR (9), but plasmid sequencing and further PCR in this study confirmed that *vagCD* was not present.

c One nucleotide was absent in one 37-bp repeat in iteron I in pE80, which might have represented a sequencing problem.

### Plasmids related to pHN7A8 producing CTX-M-65.

Plasmids pHNHNC02 (E. coli, chicken) and pHNMPC32 (E. coli, pork) showed high gene synteny with pHN7A8 (E. coli, dog), p397Kp (K. pneumoniae, human), and p477Kp (K. pneumoniae, human) (Fig. 1 and 2A). These plasmids differed by only three to eight nucleotide changes, by various numbers of 6-bp repeats in *yddA* and/or 9-bp repeats in *traD* (Table 2), and by the absence of a 2,095-bp segment (*fosA3*-*orf1*-IS*26*) on pHNMPC32 and p477Kp. The generation of a circular molecule by recombination between the two copies of IS*26* in the same orientation could lead to the insertion or loss of the *fosA3* segment.

Plasmid pHNAH33 (E. coli, chicken) was highly related to pHN7A8 but with a different arrangement of multidrug resistance region (MRR) (Fig. 1 and 2). The segments corresponding to the typical transposition unit (IS*Ecp1*-*bla*_CTX-M-65_-IS*903*-*iroN*) inserted in Tn*1722* were identical in two plasmids, with a single nucleotide change in IS*Ecp1*. The rearrangement of this segment in pHNAH33 may have arisen from IS*26*-mediated homologous recombination or transposition. In pHN7A8, the *fosA3* resistance module was followed by an incomplete Tn*2* sequence containing β-lactam resistance gene *bla*_TEM-1b_, interrupted by IS*1294* at a resolvase gene (*tnpR*). Unlike a typical mobile element, IS*1294* lacks terminal inverted repeats (TIRs), fails to generate direct repeats (DRs) of the target site, and exhibits a target site insertion with preferred tetranucleotide sequence GTTC or CTTG (19). In pHNAH33, the *fosA3* module was truncated by IS*1294*, followed by a partial Tn*2* (Δ*tnpR*-*bla*_TEM-1b_), leading to the replacement of 525 bp downstream of *fosA3*, IS*26*, and 221-bp ΔTn*2* sequences compared with pHN7A8. The insertion of an extra copy of IS*1294* at the GTTC target site within the *fosA3* module followed by homologous recombination between IS*1294* elements may have led to the deletion of the 1,566-bp region plus one IS*1294* element (Fig. 2B). On the other hand, the pHNEC55 (E. coli, pig) MRR possessed a structure related to those of pHN7A8 and pHNHNC02, but likely recombination events between IS*26* elements resulted in the acquisition of a 9,637-bp region harboring *bla*_NDM-1_ and loss of the *bla*_CTX-M-65_ module (Fig. 2A and B).

### CTX-M-55-producing plasmids pHNMPC43, pHNMPC51, pHNGD4P177, and pHNMC02 MRRs: homologous recombination in IS*26*.

Plasmids pHNGDK4P177 (pig) and pHNMC02 (chicken) were identical except for the numbers of repeats in *yddA* and *traD* (Table 2) (Fig. 1 and 2C). The pHNGD4P177 and pHNMC02 MRRs were similar to the MRR in F2:A−:B− plasmid pHK23a (GenBank accession number JQ432559) recovered from a slaughtered pig in Hong Kong (Fig. 2C) (20). A 5,847-bp region found in pHNGD4P177 and pHNMC02, containing three IS*26* elements flanking two different segments associated with parts of a 5′ conserved segment (5′-CS) and 3′-CS and the *fosA3* resistance module, was identical to that of pHK23a with opposite orientation. This observation may be associated with homologous recombination between IS*26* elements located in inverse orientations. In addition, a structure comprising a truncated *bla*_TEM-1b_ and *bla*_CTX-M-55_ within its typical (ΔIS*Ecp1*-*bla*_CTX-M-55_-*orf477*) transposition unit with a 127-bp spacer was found downstream. A similar structure was found in pHK23a with two nucleotide changes giving *bla*_CTX-M-3_ rather than *bla*_CTX-M-55_.

Plasmids pHNMPC51 and pHNMPC43 from ST35 K. pneumoniae isolates from pork showed high similarity but differed by only three nucleotide changes and repeats in *yddA* (Table 2) (Fig. 1 and 2C). The pHNMPC43/pHNMPC51 MRRs lacked the *fosA3* module and one IS*26* element compared with MRRs in pHNGD4P177/pHNMC02, which could be explained by IS*26*-mediated homologous recombination.

### Variable regions of IncN1-F33:A−:B− plasmids pHNAH9, pHNAH17, pHNFP460-1, pHNFD271, and pHN04NHB3 are closely related.

The pHNGD4P177-like plasmid MRR may have acquired an approximately 25.8-kb segment to generate the variable region of pHNAH17, as the genetic structure upstream and downstream of this ~25.8-kb segment showed 100% nucleotide identity with that found in pHNGD4P177 MRR (Fig. 3).

As a multireplicon plasmid, pHNAH17 harbored an approximately 3-kb segment (Δ*repA-*iteron I-CUP6-*resP*) corresponding to IncN1 plasmid replication region (Fig. 3). In pHNAH17, IncN replication initiation gene *repA* was truncated by IS*26* at the 5′ end, and 31 tandem repeats of 37 bp were observed within an iteron region which played an important role in determining plasmid replication and copy number control (21, 22). A 9,962-bp segment was located upstream of an IncN replication region and contained 10 putative open reading frames (ORFs). The segment shows 99% identity to a fragment of IncN plasmid pABWA45_3 (CP022157) together with the downstream IncN replication region, suggesting that pABWA45_3-like plasmids may have been the sources (Fig. 3).

An approximately 9.4-kb fragment was located downstream of an IncN region and displayed 99% identity to that of the archetypal IncI1 plasmid R64 (AP005147) backbone carrying several ORFs such as the *vagCD* addiction system (Fig. 3) (23). pHNAH17 may have acquired this segment from an IncI1 plasmid by the following two main events: (i) insertion of IS*1294* at target site 5′-GAAC into *ydjA* and (ii) transposition of IS*1294* together with the adjacent IncI1 segment by rolling-circle replication through *ori*IS to an alternative *ter*IS look-alike sequence (19) (Fig. 3). Although we failed to identify *ter*IS look-alike sequence GTTC in the 5′ end of an IncI1 fragment in pHNAH17, it is possible that a longer IncI1 segment ends in GTTC and is mobilized by IS*1294* but is truncated by IS*26* downstream of *arsR2*.

The variable region of pHNAH9 differed from that of pHNAH17 by (i) an ~25.8-kb segment in the opposite orientation that was truncated at IS*26* downstream of ΔTn*2* by insertion of IS*1294* and (ii) deletions involving the structure corresponding to IS*26*-5′CS-3′CS (Fig. 3). It suggested that the ~25.8-kb fragment was inserted into a pHNGD4P177-like MRR through IS*26* homologous recombination and IS*1294* transposition to generate pHNAH17 and that a similar insertion with the opposite orientation had occurred during pHNAH9 evolution together with IS*26*-mediated deletions.

The variable region of pHNFP460-1 was related to pHNAH9 (Fig. 3). The arrangement of the ~25.8-kb fragment was identical to that of pHNAH9 and differed by the absence of a 37-bp repeat within the iteron region (Table 2). However, IS*1294* was inserted in *pemI*/*pemK*, leading to the replacement of 53 bp of the 3′ end of *pemK* and the genetic structure (ΔIS*1*-ΔTn*2*-IS*26*). Furthermore, an approximately 8.2-kb segment consisting of three IS*26* elements flanking two different parts, located between the IncN fragment and the *fosA3* module, was present in pHNFP460-1. The first part corresponded to an additional IncN replication region, which encompassed a truncated (81-bp-shorter) *repA* gene, iteron region II with five tandem 37-bp repeats, *gshB* encoding glutathione synthetase, and 114 bp of the *ori*IS end of IS*1294*. The second part consisted of an incomplete Tn*2* and *rmtB* gene, pHNFP460-1 had an 80-bp longer Tn*2* without IS*1294* insertion than pHN7A8 and a deletion of 335 bp downstream of *rmtB*. This ~8.2-kb segment was identical to a fragment of pABWA45_3 with the exception of an IS*5075* insertion, further suggesting that pABWA45_3-like plasmids may be among the sources of IncN1-F33:A−:B− plasmids (Fig. 3).

The variable regions of pHNFD271 and pHN04NHB3 were highly similar, with five nucleotide changes (Fig. 3). As observed in IncN1-F33:A−:B− plasmids, these plasmids harbored a fragment of pABWA45_3-like plasmids without the second IncN replication region and one IS*26* element. IS*26*-mediated deletion or insertion could readily explain the observed absence or presence. In addition, an approximately 18-kb segment comprising two parts associated with IS*1294* and IS*26* was present in pHNFD271 and pHN04NHB3. The first of these (~2.9 kb) coded four putative ORFs and was found in multiple plasmids, particularly in F−:A13:B− plasmids such as pKPN528-3 (CP020856) and pK245 (DQ449578), suggesting that the F−:A13:B− plasmid may be one source. The second part (~15.1 kb) was similar (six single nucleotide polymorphisms [SNPs]) to that of IncI1 pC271 plasmids (LN735561) and exhibited 99% identity with 58% coverage to those of pHNAH9, pHNAH17, and pHNFP460-1. A sequence of approximately 50 bp downstream of the first segment in other F−:A13:B− plasmids was highly homologous to the IncI1 fragment, which might have been a site of recombination between these two parts. Therefore, this ~18-kb hybrid segment might have resulted from recombination between an IncI1 fragment and an IncFIA-like fragment. Similarly to pHNAH9 and pHNFP460-1, IS*1294*—a captured hybrid IncI1 segment together with an IncN fragment—was inserted into the *fosA3* module located in the opposite orientation at the GTTC target site, resulting in the deletion of a 1,390-bp fragment within the *fosA3* module.

The variable regions of pHNAH9, pHNAH17, pHNFP460-1, pHNFD271, and pHN04NHB3 may have been generated from pHNGD4P177-like MRR by similar insertions plus deletions or insertions of the appropriate regions.

### Variable regions of pHNZY32, pHNZY118, pHNAH24, pHNMCC14, and pHNFKU92 are closely related to those of pECB11, pE80, p42-2, and pSLK172-2.

The variable region of pHNZY32 (E. coli, patient) was the largest and consisted of six regions bounded by IS*26* or IS*1294* (Fig. 4A). The first segment corresponded to an approximately 2.8-kb structure (ΔIS*1*-ΔTn*2*-IS*26*-5′CS-3′CS), as observed in other plasmids in this study (e.g., pHNMPC43 and pHNMC02 with the same 3′-CS/IS*26* boundary).

The second segment (~15 kb) contained multiple resistance genes that included *floR* (florfenicol resistance), *tetA*/*tetR* (tetracycline resistance), *strA*/Δ*strB* (streptomycin resistance) associated with Tn*5393*, and *sul2* (sulfonamide resistance); IS*CR2* mobile elements; and incomplete Tn*5051*-like transposon Tn*AO22* (EU696790) interrupted by IS*5075* at 38-bp IR_tnp_. A similar fragment was previously detected in IncA/C plasmids lacking IS*5075*-Tn*AO22* regions such as p112298-*tetA* (KY986974, Citrobacter freundii) and pAR060302 (FJ621588, E. coli).

The third region was similar to that of pHNFD271 with an opposite orientation and contained an IncN1 pABWA45_3-like plasmid segment and the ~18-kb hybrid IncI1 segment. However, IS*5075* was inserted into a hypothetical protein in pHNZY32 and appeared to target a specific sequence similar to the 38-bp TIR of Tn*21* (Fig. 4A; see also Fig. S1 in the supplemental material). IS*5075* has been described to target a specific position in the 38-bp TIRs of Tn*21*/Tn*501* family transposons (24). Similar IS*5075* insertion was observed downstream of iteron II, further suggesting that IS*5075* displays insertion site specificity not limited to 38-bp TIR (Fig. 4A; see also Fig. S1).

10.1128/mSphere.00137-18.1 FIG S1  Insertion of IS*5075* in pHNZY32. (A) The 38-bp TIR of transposon Tn*A022* is boxed (thick lines). (B) Hypothetical protein of IncN segment. (C) Sequence downstream of iteron II. The extents of the IS*5075* sequences are indicated by bars. The IRs of IS*5075* are boxed (thin lines); arrows indicate the directions of the transposase genes. The bases in the target adjacent to which the IS*5075* inserted are shown in red, and shared bases are shown in blue. Download FIG S1, TIF file, 0.04 MB. Copyright © 2018 Wang et al. 2018 Wang et al. This content is distributed under the terms of the Creative Commons Attribution 4.0 International license.

The fourth segment (4.9 kb) contained the replication region and putative plasmid addiction system (*ddp3*-*bis*-*repX*-*pir*-*stbD*/*E*) and was identical to plasmids such as pC25 (KP722020) with the same IS*26*/IS*1294* boundary. In addition, this segment showed 98% identity to IncX1 plasmid pOLA52, although *repX* encoding a putative replication protein belonging to Rep_3 type family was absent in pOLA52 (25). It appeared that IS*1294* was inserted into an IncX1-like segment and mobilized together with the adjacent IncX1-like segment, which was truncated by IS*26*.

The fifth segment (~7.9 kb) was downstream of IS*50* and contained *aph(3′)-IIa* (aminoglycoside), Δ*ble* (bleomycin resistance), and *oqxAB* (quinolone/olaquindox resistance). *oqxAB* was flanked by two IS*26* elements in the same orientation and constituted composite transposon Tn*6010*, which was first identified in pOLA52 (26).

The last segment comprising the *fosA3* module, Δ*bla*_TEM-1b_, the typical *bla*_CTX-M-55_ transposition unit, was identical to segments in the other plasmids in this study such as pHNMC02 and pHNAH17 with the same IS*26*/IS*1* boundary.

Compared with that of pHNZY32, the variable regions of other similar plasmids differed by one nucleotide change (in pHNAH24) or three nucleotide changes (in pHNZY118, obtained from the same hospital as pHNZY32); deletions of 14 bp of the 5′ end of IS*5075* with ΔTn*AO22* downstream and one IS*5075* element (in pHNMCC14); or deletions involving the first and second IncN1 plasmid segments and an approximately 12.8-kb hybrid IncI1 segment (in pHNFKU92) (Fig. 4). IS*26*-mediated deletion may have been responsible for the creation of the similar structures present in these closely related plasmids. In addition, the latter two plasmids harbored an incomplete Tn*5393* consisting of 81-bp TIR_str_ and the entire *strA*/*strB* genes, which were identical to those of plasmids pE80, pECB11, p42-2, and pSLK172-2 but 157 bp longer than that of pHNZY32 (Fig. 4).

The variable region of pHNZY32 was related to that of pE80, except for a rearrangement, insertions of mobile elements, and deletions involving ΔTn*2* and *rmtB* (Fig. 4A and D). The *fosA3* module consisted of two parts interrupted by IS*26*, and the first part was in the opposite location compared with pHNZY32. Insertion of an extra copy of IS*26* in *orf1*, followed by homologous recombination between it and the upstream IS*26*, may explain the generation of the pE80 configuration (Fig. 4D). The pECB11 variable region similarly differed from pHNZY32 by (i) deletions involving structure (IS*26*-5′CS-3′CS) as well as a 2,157-bp segment comprising three putative ORFs and 527 bp of the *ter*IS end of IS*CR2*; (ii) the presence of an IncN region associated with partial IS*26*, located in the opposite orientation without IS*5075* insertion; and (iii) the absence of an ~47.4-kb fragment, including an IncN replication region, IncI1 segment, IncX1 segment, and *oqxAB* resistance region and the second IncN1 segment (Fig. 4E).

Previously described F33:A−:B− plasmid p42-2 from a duck and plasmid pSLK172-2 from a patient possessed pHNFKU92-related variable regions (Fig. 4F and G). p42-2 differed from pHNFKU92 by a deletion of 697 bp at the 5′ end of IS*1294* upstream of ΔIS*50* and by the presence of a 4,496-bp-longer hybrid IncI1 segment, which was truncated by IS*26* at *vagC*/*D*. The insertion site of IS*26* was the same as that of pE80, suggesting the presence of a similar IS*26* insertion in *vagC*/*D* followed by homologous recombination with another IS*26* upstream of Tn*AO22*, leading to deletions of IncN1 and part of hybrid IncI1 segments in p42-2 (Fig. 4E). Similarly to pE80, pSLK172-2 harbored an identical hybrid IncI1 segment present in p42-2. The structure of pSLK172-2 further differed from pHNFKU92 as follows: (i) the opposite locations of 5′-CS and 3′-CS segments; (ii) an IS*21* family element IS*Sso4*-like insertion in IS*1294*; and (iii) acquisition of an IncN1 replication region located between Tn*6010* and the *fosA3* module.

### Conclusion.

Comparisons of plasmids in this study to previously described F33:A−:B− plasmids revealed similar backbones and similar or distinct variable regions in E. coli isolates or K. pneumoniae of various origins from different geographic areas (mainly China). The distinct architectures of variable regions may have resulted from a series of molecular module rearrangements, acquisitions, or loss mediated by mobile elements such as IS*26* and IS*1294* via transposition or homologous recombination. We failed to observe DRs flanking IS*26* as well as specific target site duplication patterns, suggesting that IS*26*-mediated insertion, deletion, or reorganization may have occurred by homologous recombination rather than transposition (27). Common mobile elements present in F33:A−:B− plasmids, especially IS*26* and IS*1294*, not only are able to capture and mobilize antibiotic resistance genes but also are capable of acquiring fragments from other types of plasmids carrying genes involved in plasmid replication or stability or unknown functions through the process of horizontal transfer. In a similar way, other plasmids can also capture fragments of F33:A−:B− plasmids through a recombination event such as that previously observed between IncR and pHN7A8-like plasmid (28). Given that F33:A−:B− plasmids have become efficient vehicles for the dissemination of resistance genes and display the potential to capture more resistance genes or fragments from other type of plasmids through the process of evolution, their rapid spread and efficient persistence among *Enterobacteriaceae* could pose a serious threat to clinical therapy and public health.

## MATERIALS AND METHODS

### Bacterial isolates and plasmids.

A total of 17 strains carrying F33:A−:B−/IncN-F33:A−:B− plasmids were included in this study (Table 1). Isolates AHC9, AHC17, AHC24, AHC33, and HNC02 were found in chicken samples; their transconjugants/transformants carrying F33:A−:B− or IncN-F33:A−:B− plasmids were previously obtained (9). ZYTF32 and ZYTM118 were obtained from a female patient and a male patient, respectively, at the same hospital in Guangzhou, Guangdong Province, and transformants carrying IncN1-F33:A−:B− plasmids were previously described (14). Additionally, four CTX-M-55-producing E. coli strains were recovered from food-producing animals in 2009 to 2010 (29), and six CTX-M-55/65-producing E. coli or K. pneumoniae strains were isolated from food-producing animals and retail meat in Guangdong Province, China, from 2011 to 2014. For each of the 17 isolates, transformants in E. coli DH5α were obtained previously or in this study by heat-shock transformation or selected by electroporation using 2 mg/liter cefotaxime, and a single transformant of each strain that had been demonstrated to carry *bla*_CTX-M-55/-65_ and a single F33:A−:B− plasmid by PCR, sequencing, and pulsed-field gel electrophoresis with S1 nuclease was selected for further study (30, 31). MLST was performed according to previously described protocols (https://pubmlst.org/bigsdb?db=pubmlst_mlst_seqdef) (32, 33).

### Sequencing and annotation.

Seventeen plasmids were extracted from transformants using a Qiagen Plasmid Midi kit (Qiagen, Hilden, Germany). pHNFP460-1 was sequenced by the use of the Roche 454 GS-FLX platform, and contigs were assembled with 454 GS *de novo* assembler Newbler version 2.8. pHNZY32 was sequenced using PacBio single-molecule real-time sequencing (RSII platform) (Pacific Biosciences, Menlo Park, CA). Raw sequence data were introduced into the nonhybrid Hierarchical Genome Assembly Process (HGAP version 3). The remaining 15 plasmids were sequenced using Illumina MiSeq technology (Illumina, San Diego, CA). Sequence reads were assembled into contigs by the use of SOAPdenovo version 2.04.

Initial analysis and annotation of contigs were performed using the RAST server (34), ISfinder (https://www-is.biotoul.fr/), ResFinder (https://cge.cbs.dtu.dk//services/ResFinder/), RAC (http://rac.aihi.mq.edu.au/rac/), BLAST (http://blast.ncbi.nlm.nih.gov/Blast.cgi), and Gene Construction kit 4.0 (Textco BioSoftware, Inc., Raleigh, NC). Gaps between contigs were closed by PCR and Sanger sequencing. The replicon types of these plasmids were analyzed using the Plasmid MLST Database (http://pubmlst.org/plasmid/).

### Accession number(s).

The nucleotide sequences of all 17 plasmids obtained from this study were deposited in GenBank under the accession numbers listed in Table 1.
