# Supplementary material for: Evolution and Comparative Genomics of F33:A−:B− Plasmids Carrying blaCTX-M-55 or blaCTX-M-65 in Escherichia coli and Klebsiella pneumoniae Isolated from Animals, Food Products, and Humans in China
Source: mSphere. 2018 Jul 18;3(4):e00137-18. doi: 10.1128/mSphere.00137-18 (PMC6052338; doi:10.1128/mSphere.00137-18)
Supplement: TABLE S1 [file sph004182598st1.pdf]

Strain	Species	ST	Isolation time	Location	Sample origin	Plasmid	Replicon types	Reference
4B5	*E. coli*	na	2006-2008	Guangdong province, China	pet (dog or cat)	p4B5J	F33:A−:B−	1
2B1	*E. coli*	na	2006-2008	Guangdong province, China	pet (dog or cat)	p2B1J	F33:A−:B−	1
2F2	*E. coli*	na	2006-2008	Guangdong province, China	pet (dog or cat)	p2F2J	F33:A−:B−	1
4A1	*E. coli*	na	2006-2008	Guangdong province, China	pet (dog or cat)	p4A1J	F33:A−:B−	1
3D12	*K. pneumoniae*	na	2006-2008	Guangdong province, China	pet (dog or cat)	p3D12T	F33:A−:B−	1
7A8	*E. coli*	na	2006-2008	Guangdong province, China	pet (dog or cat)	p7A8T	F33:A−:B−	1
2C5	*E. coli*	na	2006-2008	Guangdong province, China	pet (dog or cat)	p2C5J	F33:A−:B−	1
0113	*E. coli*	na	2006-2008	Guangdong province, China	pet (dog or cat)	p0113-1	F33:A−:B−	1
Z426	*E. coli*	na	2006-2008	Guangdong province, China	pet (dog or cat)	pZ426-1	F33:A−:B−	1
80111	*E. coli*	na	2006-2008	Guangdong province, China	pet (dog or cat)	p80111-3	F33:A−:B−	1
LC47	*E. coli*	na	2006-2008	Guangdong province, China	pet (dog or cat)	pLC47J	F33:A−:B−	1
4E2	*E. coli*	na	2006-2008	Guangdong province, China	pet (dog or cat)	p4E2C	F33:A−:B−	1
Z425	*E. coli*	na	2006-2008	Guangdong province, China	pet (dog or cat)	pZ425-5	F33:A−:B−	1
HN4E2	*E. coli*	na	2008.5	Guangdong province, China	dog	na	F33:A−:B−	2
HN429	*E. coli*	na	2008.4	Guangdong province, China	cat	na	F33:A−:B−	2
HN2F2	*E. coli*	na	2008.5	Guangdong province, China	cat	na	F33:A−:B−	2
HN7A8*	*E. coli*	na	2008.1	Guangdong province, China	dog	pHN7A8	F33:A−:B−	2, 3
HN2B1	*E. coli*	na	2007.12	Guangdong province, China	dog	na	F33:A−:B−	2
HN3D12	*E. coli*	na	2008.4	Guangdong province, China	cat	na	F33:A−:B−	2
HN4B5	*E. coli*	na	2008.1	Guangdong province, China	dog	na	F33:A−:B−	2
HN5E3	*E. coli*	na	2008.4	Guangdong province, China	dog	na	F33:A−:B−	2
HN4A1	*E. coli*	na	2008.1	Guangdong province, China	dog	na	F33:A−:B−	2
HN127	*E. coli*	na	2010.4	Guangdong province, China	dog	na	F33:A−:B−	2
HN053	*E. coli*	na	2009.7	Guangdong province, China	dog	na	F33:A−:B−	2
HN131	*E. coli*	na	2010.5	Guangdong province, China	dog	na	F33:A−:B−	2
HN212	*E. coli*	na	2010.1	Guangdong province, China	dog	na	F33:A−:B−	2
IS75	*E. coli*	na	2010.4	Jiangxi province, China	soil	na	F33:A−:B−	4
IS89a	*E. coli*	na	2010.4	Jiangxi province, China	soil	na	F33:A−:B−	4
IS90a	*E. coli*	na	2010.4	Jiangxi province, China	soil	na	F33:A−:B−	4
OS43	*E. coli*	na	2010.4	Jiangxi province, China	soil	na	F33:A−:B−	4
BP11	*E. coli*	na	2010.4	Jiangxi province, China	boar	na	F33:A−:B−	4
SP38	*E. coli*	na	2010.4	Jiangxi province, China	sow	na	F33:A−:B−	4
SP28	*E. coli*	na	2010.4	Jiangxi province, China	sow	na	F33:A−:B−	4

Strain	Species	ST	Isolation time	Location	Sample origin	Plasmid	Replicon types	Reference
SP32	*E. coli*	na	2010.4	Jiangxi province, China	sow	na	F33:A−:B−	4
SP17	*E. coli*	na	2010.4	Jiangxi province, China	sow	na	F33:A−:B−	4
SP26	*E. coli*	na	2010.4	Jiangxi province, China	sow	na	F33:A−:B−	4
SP35	*E. coli*	na	2010.4	Jiangxi province, China	sow	na	F33:A−:B−	4
SP25	*E. coli*	na	2010.4	Jiangxi province, China	sow	na	F33:A−:B−	4
SP39	*E. coli*	na	2010.4	Jiangxi province, China	sow	na	F33:A−:B−	4
IS84b	*Enterobacter aerogenes*	na	2010.4	Jiangxi province, China	soil	na	F33:A−:B−	4
ZGL74	*E. coli*	410	2008.2	Guangdong province, China	pigeon	na	F33:A−:B−	5
SB1037	*E. coli*	354	2008.9	Guangdong province, China	chicken	na	F33:A−:B−	5
42-2	*E. coli*	na	2010	Guangdong province, China	duck	p42-2	F33:A−:B−	6, 7
FS5E1D	*E. coli*	na	2012	Guangdong province, China	goose	na	F33:A−:B−	6
FS9Y1C	*E. coli*	na	2012	Guangdong province, China	duck	na	IncN-F33:A−:B−	6
FS2Y1X	*E. coli*	na	2012	Guangdong province, China	duck	na	F33:A−:B−	6
AHC9*	*E. coli*	48	2011.6	Anhui province, China	chicken	na	IncN-F33:A-:B-	8
AHC17*	*E. coli*	4483	2011.6	Anhui province, China	chicken	na	IncN-F33:A−:B−	8
AHC18	*E. coli*	4447	2011.6	Anhui province, China	chicken	na	F33:A−:B−	8
AHC23	*E. coli*	2607	2011.6	Anhui province, China	chicken	na	F33:A−:B−	8
AHC24*	*E. coli*	155	2011.6	Anhui province, China	chicken	na	IncN-F33:A−:B−	8
AHC26	*E. coli*	23	2011.6	Anhui province, China	chicken	na	IncN-F33:A−:B−	8
AHC33*	*E. coli*	101	2011.6	Anhui province, China	chicken	na	F33:A−:B−	8
AHC52	*E. coli*	206	2011.7	Anhui province, China	chicken	na	IncN-F33:A−:B−	8
AHC67	*E. coli*	2223	2011.7	Anhui province, China	chicken	na	F33:A−:B−	8
AHC69	*E. coli*	2847	2011.7	Anhui province, China	chicken	na	F33:A−:B−	8
AHC72	*E. coli*	2847	2011.7	Anhui province, China	chicken	na	F33:A−:B−	8
GDC1-4	*E. coli*	453	2010.9	Guangdong province, China	chicken	na	F33:A−:B−	8
GDC17	*E. coli*	1518	2010.8	Guangdong province, China	chicken	na	IncN-F33:A−:B−	8
GDC24	*E. coli*	4477	2010.8	Guangdong province, China	chicken	na	F33:A−:B−	8
GDC40	*E. coli*	744	2010.8	Guangdong province, China	chicken	na	F33:A−:B−	8
GDC46	*E. coli*	4460	2010.8	Guangdong province, China	chicken	na	F33:A−:B−	8
GDC47	*E. coli*	746	2010.8	Guangdong province, China	chicken	na	F33:A−:B−	8
GDC54	*E. coli*	2496	2010.8	Guangdong province, China	chicken	na	F33:A−:B−	8
GDC58	*E. coli*	4461	2010.8	Guangdong province, China	chicken	na	F33:A−:B−	8

Strain	Species	ST	Isolation time	Location	Sample origin	Plasmid	Replicon types	Reference
GDC114	*E. coli*	48	2010.9	Guangdong province, China	chicken	na	F33:A−:B−	8
GDC240	*E. coli*	354	2010.8	Guangdong province, China	chicken	na	F33:A−:B−	8
HNC02*	*E. coli*	4464	2009.7	Henan province, China	chicken	na	F33:A−:B−	8
SDC13	*E. coli*	398	2009.5	Shandong province, China	chicken	na	F33:A−:B−	8
SDC04	*E. coli*	602	2009.5	Shandong province, China	chicken	na	F33:A−:B−	8
SDC01	*E. coli*	4462	2009.5	Shandong province, China	chicken	na	IncN-F33:A−:B−	8
85CHANG YI	*E. coli*	na	2010	Guangdong province, China	duck	na	F33:A−:B−	9
38	*E. coli*	na	2010	Guangdong province, China	dog	na	F33:A−:B−	10
50	*E. coli*	na	2010	Guangdong province, China	cat	na	F33:A−:B−	10
65	*E. coli*	na	2010	Guangdong province, China	dog	na	F33:A−:B−	10
D8	*E. coli*	na	2010	Guangdong province, China	dog	na	F33:A−:B−	10
FS6J1W	*E. coli*	na	2012	Guangdong province, China	avian (duck, chicken, geese)	na	F33:A−:B−	11
FS8J4C	*E. coli*	na	2012	Guangdong province, China	avian (duck, chicken, geese)	na	F33:A−:B−	11
FS6Y1G	*E. coli*	na	2012	Guangdong province, China	avian (duck, chicken, geese)	na	F33:A−:B−	11
FS2Y3G	*E. coli*	na	2012	Guangdong province, China	avian (duck, chicken, geese)	na	F33:A−:B−	11
NND6	*E. coli*	na	2010	Guangdong province, China	avian (duck, chicken, geese)	na	F33:A−:B−	11
FS4Y2C	*E. coli*	na	2012	Guangdong province, China	avian (duck, chicken, geese)	na	F33:A−:B−	11
2YC2	*E. coli*	na	2011	Guangdong province, China	avian (duck, chicken, geese)	na	F33:A−:B−	11
FS11Y2G	*E. coli*	na	2012	Guangdong province, China	avian (duck, chicken, geese)	na	F33:A−:B−	11
FS11Y2G	*E. coli*	na	2012	Guangdong province, China	avian (duck, chicken, geese)	na	F33:A−:B−	11
FS6Y4C	*E. coli*	na	2012	Guangdong province, China	avian (duck, chicken, geese)	na	F33:A−:B−	11
S55-2	*E. coli*	na	2012	Guangdong province, China	pig	na	F33:A−:B−	11
FS1Z1X	*E. coli*	na	2012	Guangdong province, China	pig	na	F33:A−:B−	11
FS11Z5F	*E. coli*	na	2012	Guangdong province, China	pig	na	F33:A−:B−	11
FS13Z2C	*E. coli*	na	2012	Guangdong province, China	pig	na	F33:A−:B−	11
FS13Z3C	*E. coli*	na	2012	Guangdong province, China	pig	na	F33:A−:B−	11
3-3-2	*E. coli*	na	2008	Guangdong province, China	pig	na	F33:A−:B−	11
S104	*E. coli*	na	2012	Guangdong province, China	pig	na	F33:A−:B−	11
FS5Z6D	*E. coli*	na	2012	Guangdong province, China	pig	na	F33:A−:B−	11
S68	*E. coli*	na	2010	Guangdong province, China	pig	na	F33:A−:B−	11
1-5-1	*E. coli*	na	2008	Guangdong province, China	pig	na	F33:A−:B−	11
P161	*E. coli*	na	2008	Guangdong province, China	pet (dog or cat)	na	F33:A−:B−	11
B7	*E. coli*	na	2010	Guangdong province, China	pet (dog or cat)	na	F33:A−:B−	11

Strain	Species	ST	Isolation time	Location	Sample origin	Plasmid	Replicon types	Reference
E80	*E. coli*	ND	2013	Guangdong province, China	chicken meat	pE80	IncN-F33:A−:B−	12
THSJ02	*E. coli*	167	2014	Guangdong province, China	chicken meat	na	F33:A−:B−	13
ZYTF32*	*E. coli*	58	2013	Guangdong province, China	patient	na	IncN-F33:A−:B−	14
ZYTM118*	*E. coli*	NEW	2013	Guangdong province, China	patient	na	IncN-F33:A−:B−	14
ZYTF154	*E. coli*	NEW	2013	Guangdong province, China	patient	na	IncN-F33:A−:B−	14
HNEC55	*E. coli*	1721	2014 or 2015	Henan province, China	pig	pHNEC55	F33:A−:B−	15
HNEC46	*E. coli*	1695	2014 or 2015	Henan province, China	pig	pHNEC46	F33:A−:B−	15
SLK172	*E. coli*	189	2015	Beijing, China	patient	pSLK172-2	F33:A−:B−	16
SY303P	*E. coli*	3315	2015 or 2016	Liaoning province, China	pig	na	IncN-F33:A−:B−	17
SY301L	*E. coli*	5442	2015 or 2016	Liaoning province, China	pig	na	F33:A−:B−	17
CH293B	*E. coli*	10	2015 or 2016	Jilin province, China	chicken	na	F33:A−:B−	17
CH292B	*E. coli*	10	2015 or 2016	Jilin province, China	chicken	pECB11	F33:A−:B−	17
CH291M	*E. coli*	410	2015 or 2016	Jilin province, China	chicken	na	IncN-F33:A−:B−	17
DH286F	*E. coli*	2518	2015 or 2016	Jilin province, China	chicken	pECF12	F33:A−:B−	17
DH286M	*E. coli*	617	2015 or 2016	Jilin province, China	chicken	na	F33:A−:B−	17
JL12G	*E. coli*	559	2015 or 2016	Jilin province, China	cow	na	F33:A−:B−	17
JL15P	*E. coli*	209	2015 or 2016	Jilin province, China	cow	na	F33:A−:B−	17
HL12L	*E. coli*	195	2015 or 2016	Innner Mongolia autonomous region, China	cow	na	F33:A−:B−	17
HB37B	*E. coli*	1725	2015 or 2016	Heilongjiang province, China	chicken	na	F33:A−:B−	17
HB13B	*E. coli*	167	2015 or 2016	Heilongjiang province, China	chicken	na	F33:A−:B−	17
SH312M	*E. coli*	1488	2015 or 2016	Heilongjiang province, China	pig	na	F33:A−:B−	17
SH21F	*E. coli*	209	2015 or 2016	Heilongjiang province, China	pig	na	F33:A−:B−	17
SH21G	*E. coli*	354	2015 or 2016	Heilongjiang province, China	pig	na	F33:A−:B−	17
SH21M	*E. coli*	648	2015 or 2016	Heilongjiang province, China	pig	na	F33:A−:B−	17
SC-F3-26	*E. coli*	NEW	2015	Sichuan province, China	chicken	pSC-F3-26	F33:A−:B−	18
SC-F6-3	*E. coli*	4980	2014	Sichuan province, China	chicken	pSC-F6-3	F33:A−:B−	18
SC-F1-7	*E. coli*	359	2015	Sichuan province, China	chicken	pSC-F1-7	F33:A−:B−	18
NX-4	*E. coli*	NEW	2015	Ningxia province, China	chicken	pNX-4	F33:A−:B−	18
SC-F6-2	*E. coli*	NEW	2014	Sichuan province, China	chicken	pSC-F6-2	F33:A−:B−	18
AH-4	*E. coli*	NEW	2015	Anhui province, China	chicken	pAH-4	F33:A−:B−	18
NX-10	*E. coli*	NEW	2015	Ningxia province, China	chicken	pNX-10	F33:A−:B−	18
TJ-14	*E. coli*	457	2015	Tianjian, China	chicken	pTJ-14	F33:A−:B−	18

Strain	Species	ST	Isolation time	Location	Sample origin	Plasmid	Replicon types	Reference
KP07	*K. pneumoniae*	37	2012	Guangdong province, China	dog	na	IncN-F33:A−:B−	19
KP21	*K. pneumoniae*	37	2012	Guangdong province, China	dog	na	IncN-F33:A−:B−	19
KP22	*K. pneumoniae*	37	2012	Guangdong province, China	dog	na	IncN-F33:A−:B−	19
KP23	*K. pneumoniae*	37	2012	Guangdong province, China	dog	na	IncN-F33:A−:B−	19
KP24	*K. pneumoniae*	37	2012	Guangdong province, China	dog	na	IncN-F33:A−:B−	19
KP25	*K. pneumoniae*	37	2012	Guangdong province, China	dog	na	IncN-F33:A−:B−	19
KP26	*K. pneumoniae*	37	2012	Guangdong province, China	dog	na	IncN-F33:A−:B−	19
KP29	*K. pneumoniae*	37	2012	Guangdong province, China	dog	na	IncN-F33:A−:B−	19
KP30	*K. pneumoniae*	37	2012	Guangdong province, China	dog	na	IncN-F33:A−:B−	19
397Kp	*K. pneumoniae*	726	2013	Bolivia	patient	p397Kp	F33:A−:B−	20
477Kp	*K. pneumoniae*	726	2013	Bolivia	patient	p477Kp	F33:A−:B−	20
FA10	*E. coli*	457	2013-2016	Brazil	chicken	na	F33:A−:B−	21
FA20	*E. coli*	457	2013-2016	Brazil	chicken	na	F33:A−:B−	21
FA23	*E. coli*	457	2013-2016	Brazil	chicken	na	F33:A−:B−	21
FA27	*E. coli*	453	2013-2016	Brazil	chicken	na	F33:A−:B−	21
FA46	*E. coli*	117	2013-2016	Brazil	chicken	na	F33:A−:B−	21
FA50	*E. coli*	1706	2013-2016	Brazil	chicken	na	F33:A−:B−	21

Strains carrying F33:A−:B− were used in this study were indicated by an asterisk. Strains obtained in our previous research (reference 4) were described in East China, we specified it here as Jiangxi province.

References

1 **Deng Y, He L, Chen S, Zheng H, Zeng Z, Liu Y, Sun Y, Ma J, Chen Z, Liu JH.** 2011. F33:A-:B- and F2:A-:B- plasmids mediate dissemination of *rmtB-bla*$_{CTX-M-9}$ group genes and *rmtB-qepA* in Enterobacteriaceae isolates from pets in China. Antimicrob Agents Chemother **55**: 4926-4629. http://dx.doi.org/10.1128/AAC.00133-11.

2 **Hou J, Huang X, Deng Y, He L, Yang T, Zeng Z, Chen Z, Liu JH.** 2012. Dissemination of the fosfomycin resistance gene *fosA3* with CTX-M β-lactamase genes and *rmtB* carried on IncFII plasmids among *Escherichia coli* isolates from pets in China. Antimicrob Agents Chemother **56**: 2135-2138. http://dx.doi.org/10.1128/AAC.05104-11.

3 **He L, Partridge SR, Yang X, Hou J, Deng Y, Yao Q, Zeng Z, Chen Z, Liu JH.** 2013. Complete nucleotide sequence of pHN7A8, an F33:A-:B-type epidemic plasmid carrying *bla*$_{CTX-M-65}$, *fosA3* and *rmtB* from China. J Antimicrob Chemother **68**: 46-50. http://dx.doi.org/10.1093/jac/dks369.

4 **Yao Q, Zeng Z, Hou J, Deng Y, He L, Tian W, Zheng H, Chen Z, Liu JH.** 2011. Dissemination of the *rmtB* gene carried on IncF and IncN plasmids among Enterobacteriaceae in a pig farm and its environment. J Antimicrob Chemother **66**: 2475-2479. http://dx.doi.org/10.1093/jac/dkr328.

5 **Hou J, Yang X, Zeng Z, Lv L, Yang T, Lin D, Liu JH.** 2013. Detection of the plasmid-encoded fosfomycin resistance gene *fosA3* in *Escherichia coli* of food-animal origin. J Antimicrob Chemother **68**: 766-770. http://dx.doi.org/10.1093/jac/dks465.

6 **Liu BT, Yang QE, Li L, Sun J, Liao XP, Fang LX, Yang SS, Deng H, Liu YH**. 2013. Dissemination and characterization of plasmids carrying *oqxAB-bla*$_{CTX-M}$ genes in *Escherichia coli* isolates from food-producing animals. PLoS One **8**: e73947. http://dx.doi.org/10.1371/journal.pone.0073947.

7 **Yang QE, Walsh TR, Liu BT, Zou MT, Deng H, Fang LX, Liao XP, Sun J, Liu YH.** 2016. Complete sequence of the FII plasmid p42-2, carrying *bla*$_{CTX-M-55}$, *oqxAB*, *fosA3*, and *floR* from *Escherichia coli*. Antimicrob Agents Chemother **60**: 4336-4338. http://dx.doi.org/10.1128/AAC.00475-16.

8 **Yang X, Liu W, Liu Y, Wang J, Lv L, Chen X, He D, Yang T, Hou J, Tan Y, Xing L, Zeng Z, Liu JH.** 2014. F33:A-:B-, IncHI2/ST3, and IncI1/ST71 plasmids drive the dissemination of *fosA3* and *bla*$_{CTX-M-55/-14/-65}$ in *Escherichia coli* from chickens in China. Front Microbiol **5**: 688. http://dx.doi.org/10.3389/fmicb.2014.00688.

9 **Liu BT, Li L, Fang LX, Sun J, Liao XP, Yang QE, Huang T, Liu YH.** 2014. Characterization of plasmids carrying *oqxAB* in *bla*$_{CTX-M}$-negative *Escherichia coli* isolates from food-producing animals. Microb Drug Resist **20**: 641-50. http://dx.doi.org/ 10.1089/mdr.2014.0022.

10 **Liao XP, Liu BT, Yang QE, Sun J, Li L, Fang LX, Liu YH**. 2013. Comparison of plasmids coharboring 16s rrna methylase and extended-spectrum β-lactamase genes among Escherichia coli isolates from pets and poultry. J Food Prot **76**: 2018-23. http://dx.doi.org/10.4315/0362-028X.JFP-13-200.

11 **Yang QE, Sun J, Li L, Deng H, Liu BT, Fang LX, Liao XP, Liu YH**. 2015. IncF plasmid diversity in multi-drug resistant *Escherichia coli* strains from animals in China. Front Microbial **6**: 964. http://dx.doi.org/10.3389/fmicb.2015.00964.

12 **Wong MH, Xie M, Xie L, Lin D, Li R, Zhou Y, Chan EW, Chen S.** 2016. Complete sequence of a F33:A-:B- conjugative plasmid carrying the *oqxAB*, *fosA3*, and $bla_{\text{CTX-M-55}}$ elements from a foodborne *Escherichia coli* strain. Front Microbiol **7**: 1729. http://dx.doi.org/10.3389/fmicb.2016.01729.

13 **Yao X, Doi Y, Zeng L, Lv L, Liu JH.** 2016. Carbapenem-resistant and colistin-resistant *Escherichia coli* co-producing NDM-9 and MCR-1. Lancet Infect Dis **16**:288-289. http://dx.doi.org/10.1016/S1473-3099(16)00057-8.

14 **Wang J, Zhi CP, Chen XJ, Guo ZW, Liu WL, Luo J, Huang XY, Zeng L, Xia YB, Yi MY, Huang T, Zeng ZL, Liu JH.** 2017. Characterization of *oqxAB* in *Escherichia coli* isolates from animals, retail meat, and human patients in Guangzhou, China. Front Microbiol **8**: 1982. http://dx.doi.org/10.3389/fmicb.2017.01982.

15 **Lin D, Xie M, Li R, Chen K, Chan EW, Chen S**. 2016. IncFII conjugative plasmid-mediated transmission of $bla_{\text{NDM-1}}$ elements among animal-borne *Escherichia coli* strains. Antimicrob Agents Chemother **61**: e02285-16. http://dx.doi.org/10.1128/AAC.02285-16.

16 **Bai L, Wang J, Hurley D, Yu Z, Wang L, Chen Q, Li J, Li F, Fanning S.** 2017. A novel disrupted *mcr-1* gene and a lysogenized phage P1-like sequence detected from a large conjugative plasmid, cultured from a human atypical enteropathogenic *Escherichia coli* (aEPEC) recovered in China. J Antimicrob Chemother **72**: 1531-1533. http://dx.doi.org/10.1093/jac/dkw564.

17 **Wang XM, Dong Z, Schwarz S, Zhu Y, Hua X, Zhang Y, Liu S, Zhang WJ.** 2017. Plasmids of diverse Inc groups disseminate the fosfomycin resistance gene *fosA3* among *Escherichia coli* from pigs, chickens and dairy cows in Northeast China. Antimicrob Agents Chemother **61**. e00859-17. http://dx.doi.org/10.1128/AAC.00859-17.

18 **Jiang W, Men S, Kong L, Ma S, Yang Y, Wang Y, Yuan Q, Cheng G, Zou W, Wang H.** 2017. Prevalence of plasmid-mediated fosfomycin resistance gene *fosA3* among CTX-M-producing *Escherichia coli* isolates from chickens in China. Foodborne Pathog Dis **14**:210-218. https://doi.org/10.1089/fpd.2016.2230.

19 **Xia J, Fang LX, Cheng K, Xu GH, Wang XR, Liao XP, Liu YH, Sun J.** 2017. Clonal spread of 16S rRNA methyltransferase-producing *Klebsiella pneumoniae* ST37 with high prevalence of ESBLs from companion animals in China. Front Microbiol **8**:529. https://doi.org/10.3389/fmicb.2017.00529.

20 **Sennati S, Riccobono E, Di Pilato V, Villagran AL, Pallecchi L, Bartoloni A, Rossolini GM.** 2016. pHN7A8-related multiresistance plasmids ($bla_{\text{CTX-M-65}}$, *fosA3* and *rmtB*) detected in clinical isolates of *Klebsiella pneumoniae* from Bolivia: intercontinental plasmid dissemination? J Antimicrob Chemother **71**: 1732-1734. http://dx.doi.org/10.1093/jac/dkv506.

21 Cunha MP, Lincopan N, Cerdeira L, Esposito F, Dropa M, Franco LS, Moreno AM, Knöbl T. 2017. Coexistence of CTX-M-2, CTX-M-55, CMY-2, FosA3, and QnrB19 in extraintestinal pathogenic *Escherichia coli* from poultry in Brazil. Antimicrob Agents Chemother **61**. pii: e02474-16. http://dx.doi.org/10.1128/AAC.02474-16.
