# Supplementary figures and images for: Evolution and Comparative Genomics of F33:A−:B− Plasmids Carrying blaCTX-M-55 or blaCTX-M-65 in Escherichia coli and Klebsiella pneumoniae Isolated from Animals, Food Products, and Humans in China
Source: mSphere. 2018 Jul 18;3(4):e00137-18. doi: 10.1128/mSphere.00137-18 (PMC6052338; doi:10.1128/mSphere.00137-18)

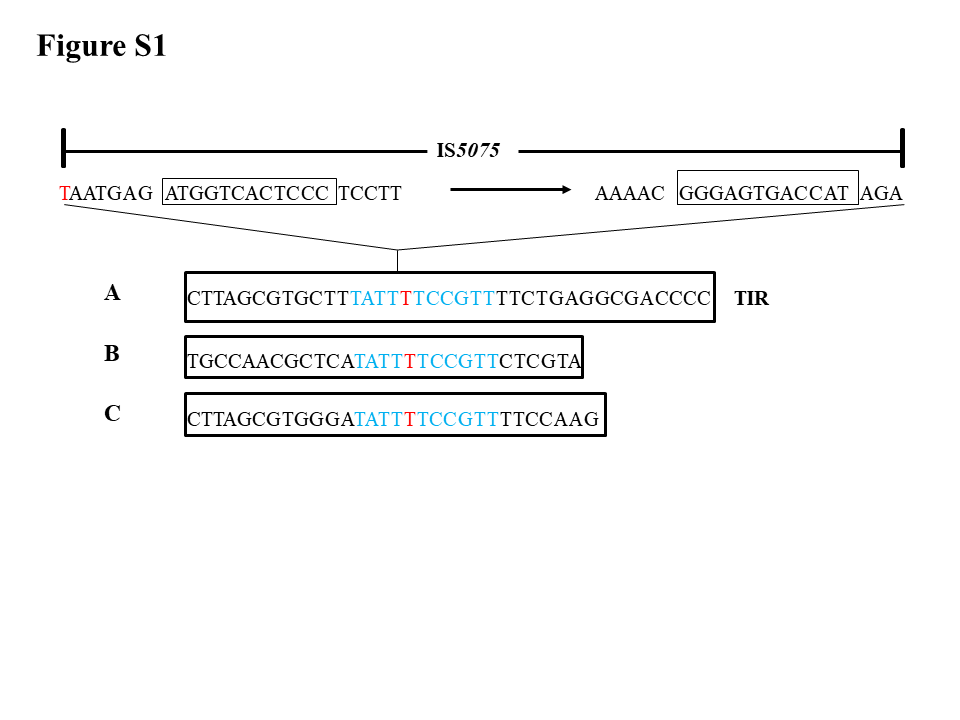

Supplement: FIG S1 [file sph004182598sf1.tif]
